# gwid: an R package and Shiny application for Genome-Wide analysis of IBD data

**DOI:** 10.1093/bioadv/vbae115

**Published:** 2024-07-31

**Authors:** Soroush Mahmoudiandehkordi, Mehdi Maadooliat, Steven J Schrodi

**Affiliations:** Department of Mathematical and Statistical Sciences, Marquette University, Milwaukee, WI 53233, United States; Department of Medical Genetics, University of Wisconsin-Madison, Madison, WI 53706, United States; Department of Mathematical and Statistical Sciences, Marquette University, Milwaukee, WI 53233, United States; Department of Medical Genetics, University of Wisconsin-Madison, Madison, WI 53706, United States; Department of Medical Genetics, University of Wisconsin-Madison, Madison, WI 53706, United States; Computation and Informatics in Biology and Medicine, University of Wisconsin-Madison, Madison, WI 53706, United States

## Abstract

**Summary:**

Genome-wide identity by descent (gwid) is an R package developed for the analysis of identity-by-descent (IBD) data pertaining to dichotomous traits. This package offers a set of tools to assess differential IBD levels for the two states of a binary trait, yielding informative and meaningful results. Furthermore, it provides convenient functions to visualize the outcomes of these analyses, enhancing the interpretability and accessibility of the results. To assess the performance of the package, we conducted an evaluation using real genotype data derived from the SNPs to investigate rheumatoid arthritis susceptibility from the Marshfield Clinic Personalized Medicine Research Project.

**Availability and implementation:**

gwid is available as an open-source R package. Release versions can be accessed on CRAN (https://cran.r-project.org/package=gwid) for all major operating systems. The development version is maintained on GitHub (https://github.com/soroushmdg/gwid) and full documentation with examples and workflow templates is provided *via* the package website (http://tinyurl.com/gwid-tutorial). An interactive R Shiny dashboard is also developed (https://tinyurl.com/gwid-shiny).

## 1 Introduction

Identity-by-descent (IBD) mapping is a powerful approach for investigating the genetic underpinnings of complex diseases. When two haplotypes inherit identical alleles from a recent shared ancestor, they are considered identical-by-descent. Individuals with recent common ancestry (approximately 25 generations) are likely to have overlapping chromosomal regions due to this phenomenon (Thompson 2013). Rare causal alleles, which exert more significant effects than common causal alleles, can often be traced back to recent ancestry, as natural selection tends to eliminate deleterious rare variants from a population. This mapping technique, coupled with disease genetics models involving multiple variants contributing to disease susceptibility within specific chromosomal regions, is particularly suitable for rare alleles with substantial effects. Moreover, this approach is adept at identifying dominant effects. Hence, whereas traditional genome-wide association studies (GWAS) analyses are well-powered to detect additive effects from alleles segregating at a higher frequency, IBD mapping offers advantages for detecting chromosomal regions linked to disease-carrying rare, highly penetrant alleles which can exhibit non-additive modes of inheritance. Further, through capturing a linkage signal, non-interrogated disease alleles within a chromosomal region can be indirectly detected even if they reside outside a region of linkage disequilibrium (LD) as measured by non-related population-based samples. It is advantageous to employ this method in genetically homogeneous populations, such as large extended kinships or isolated groups, where many affected individuals may have inherited a disease-associated haplotype from a recent ancestor (Browning and Browning 2012).

Most of the existing algorithms for disease gene mapping through association have a strong assumption of independence between subjects (Balding 2006, Marees *et al.* 2018). This assumption is often untenable, particularly for isolated populations. Therefore, statistical techniques that capture and incorporate the dependence structure among the subjects will provide more efficient and accurate results. The intricacy of the matter becomes notably demanding when dealing with IBD data, as each recorded observation signifies the inferred IBD status between two individuals at a specific genomic location. Furthermore, it should be acknowledged that these individuals may also display IBD relationships with other individuals, or with themselves, at different genomic loci. Consequently, it becomes imperative to tackle this inherent lack of independence among pairwise individuals in IBD data. Appropriate statistical assessment and harnessing of the relatedness structure in sample sets to increase the power to detect disease-predisposing regions of the genome is critically important as most commonly used biobanks—such as the UK Biobank, FINNGEN, China Kadoorie Biobank, and Estonian Biobank—are known to exhibit substantial shared IBD patterns among the subjects (Nait Saada *et al.* 2020, Pankratov *et al.* 2020, Kurki *et al.* 2023, Walters *et al.* 2023).

In this pipeline, we have developed the genome-wide identity by descent (gwid) R package, with a primary focus on the analysis of IBD data to interrogate chromosomal regions that are linked and associated with a dichotomous trait or disease (similar to Browning and Thompson 2012, approach, which compares case/case and case/control pairwise subjects in population-based samples, e.g. WTCCC). The operating hypothesis is that statistically significant regions likely house one or more susceptibility variants. This library effectively incorporates functions from established R packages to model the evaluation of IBD-phenotype association. Acting as a wrapper, our software empowers users to conduct analyses on a group of SNPs and their associated IBD regions, while allowing for the specification of user-defined parameters. Moreover, as gwid employs an object-oriented programming approach, it can automatically produce a comprehensive and user-friendly summary of both the results and plots. This is made possible through a limited set of functions and methods that contribute to a clear and convenient understanding of the findings.

**Figure 1.**
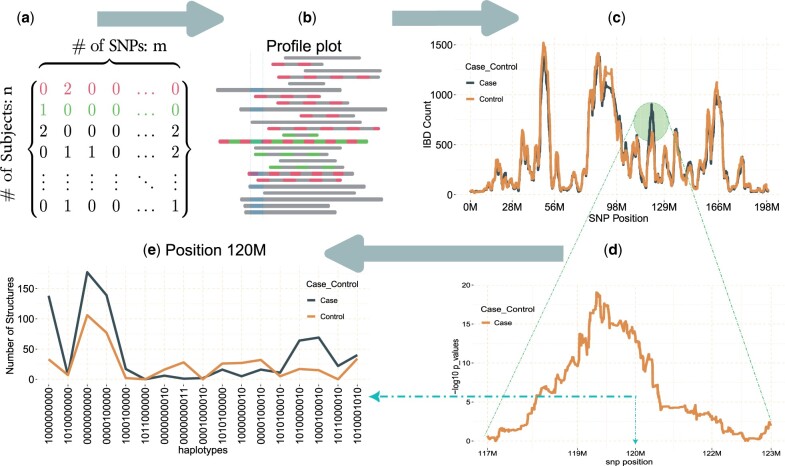
gwid pipeline: (a) The schema of SNP panel data, depicting *n* SNP variants across *m* samples. The digits 0, 1, and 2 typically indicate the number of minor allele copies and two subjects with tracked IBD segments are highlighted in the profile plot shown in part b. (b) The pairwise IBD relationships within the SNP data are represented. Schematic representation of IBD sharing between the highlighted subjects, both among themselves and in relation to other individuals, illustrated within the specified window. (c) Frequency of IBD events among case and control subjects as exemplified by the RA study, with counts provided and both groups comprising an equal number of participants. A pronounced elevation in the quantity of IBD in the case group becomes visually apparent between the genomic positions of 117M and 123M. (d) Screenshot of the Fisher exact test to compare counts of controls with cases. (e) The panel showcases the haplotype counts, in case and control subjects, specifically using a window of size 10 SNPs at the 120M location.

One advantage of the proposed method is that imputation is typically not needed, though it can enhance precision. However, for cases where genotype imputation is necessary, researchers may utilize the Michigan Imputation Server (Das *et al.* 2016), ensuring that the sample’s ancestral background is compatible with the selected reference sequences. Modern SNP (single nucleotide polymorphism) arrays (Fig. 1a) provide adequate density for accurately defining IBD regions. For example, for the low end of IBD lengths in 5th-degree relatives, which is around 1–2 Mb in length, SNP arrays with a density of approximately 250 000 markers should yield around 50–100 SNPs within that region. This coverage is sufficient for identifying IBD regions in somewhat distantly related individuals, classified as extended kinship. Thus, while low-density SNP arrays may suffice in certain scenarios, higher density arrays may be necessary in others. Furthermore, for comprehensive analysis, one might prefer whole-genome sequencing compared to exome sequencing, which provides full genomic coverage. Alternatively, conducting a genome-wide SNP scan followed by IBD analysis and targeted sequencing of potentially functional rare variants in coding regions could be more informative for the traits under study. One may note that, this approach may not be ideal for samples from multiple populations or those with recent gene flow into the population. For long IBD regions, cases and controls sharing the same IBD region have similar genetic ancestry, which mitigates the issue of population stratification. However, ancestry-specific interaction effects between the IBD region and other genomic areas can still occur. To minimize these effects, it is crucial to identify individuals with similar global ancestry across the genome. To address this challenge, applying PCA to genotyped samples, especially those with self-reported or unknown ancestry labels, can enhance the accuracy of ancestry estimation, thereby helping to identify individuals from genetically homogeneous populations.

## 2 Methods

### 2.1 Data description

The pipeline needs three input files: (i) an IBD file, (ii) a genotype file, and (iii) a phenotype file. Various software tools have been developed to estimate IBD segments, including King, RaPID, Phaseibd, FastSMC, and IBIS. In our package, we utilized Refined-IBD (Browning and Browning 2013, Browning *et al.* 2018, 2021), but any of these models can be employed if their output data structures are adapted to match the format used by Refined-IBD. The IBD file should consist of a data frame that includes information such as the first sample identifier, first sample haplotype index (1 or 2), second sample identifier, second sample haplotype index (1 or 2), chromosome, starting genomic position, and ending genomic position. The Refined-IBD output includes two additional columns: the LOD score (logarithm of odds) and the cM length of the IBD segment. However, we did not utilize these columns in the gwid package.

For each chromosome, it is required that the genotype file be in the form of a GDS file. A data storage format designed for the efficient handling and quick random access to specific segments of genomic information. For more information on GDS read (Zheng *et al.* 2012). The phenotype file, in the form of an R data file organized as a list, should contain the individual ID and the phenotype of interest, specifically the case-control status for each ID.

For users interested in extracting haplotypes over specified window sizes, a phased haplotype data file is necessary. The required file format is VCF, which is generated as output from the Beagle software (Browning *et al.* 2021).

### 2.2 The gwid workflow for investigating disease linkage/association through IBD

The pipeline initially converts the IBD data (Fig. 1b) into a sparse matrix, where each row represents either pairwise case-case or control-control individuals (we refer to these as case group and control group for simplicity), and each column corresponds to a genetic location. The observations in the matrix are binary, with 1 indicating IBD and 0 indicating non-IBD between pairwise individuals. Furthermore, the package utilizes the frequencies of IBD within the different groups as a basis for comparison at each genetic location (Fig. 1c). If the frequency of IBD is higher in the case group compared to the control group, it suggests an association and linkage between that region and the disease.

The plot method can be applied to an object of the gwid class in two ways: firstly, to create profile plots (Fig. 1b), aiding in the visualization of IBD segments either throughout the entire genome or in specified areas, thereby displaying the IBD counts for each group; secondly, to analyse IBD frequency across the genome in a sample set and to pinpoint varying frequencies among subject groups (Fig. 1c).

The package employs statistical analyses to assess the linkage and association between IBD regions and disease susceptibility. It quantifies the disparity in IBD occurrences for individual SNPs within autosomal regions among both pairwise subject groups. fisher_test() method executes the Fisher’s exact test to detect a potential association between distinct genetic loci and the disease. To determine the significant threshold, Li and Ji (2005) proposed a method that calculate the eigenvalues of the correlation matrix, which is based on mutual information rather than Pearson correlation, to estimate the effective number of independent tests. This estimate is then used within the Benjamini and Hochberg (1995) procedure to control the false discovery rate (FDR) and establish the significance threshold. On the other hand, as pairs of individuals exhibiting shared IBD regions can overlap with other individuals therefore violating the independence assumption of Fisher’s exact test, a permutation-based *P* values can also be computed using permutation_test() method.

This IBD based approach has exhibited notable efficacy in contrast to conventional GWAS, which might be limited in detecting rare variants or combinations of predisposing variants within a chromosomal region. To compare IBD results to traditional association analyses, the package can also perform a GWAS analysis using input SNP panel data (Fig. 1a). This is useful for comparing and supplementing association-based signals produced by higher frequency alleles and those results obtained from IBD. fisher_test() method can use Fisher’s exact test on individual SNP data to analyse binary traits. Also, users can run permutation tests alongside Fisher’s exact test.

The output of the gwid provides a concise summary of results, including *P* values for each test conducted for every SNP, which can be further analysed. Additionally, the plot() method can be used to visualize these *P* values across the genome, aiding in the understanding of the data structure, identification of causal regions, and detection of any artifacts (Fig. 1d).

### 2.3 The incorporation of sliding window approach to account for signal from multiple SNPs

The library extends its capabilities to encompass the analysis of IBD data through the inclusion of a generalized method that allows for the incorporation of neighboring SNPs. This generalization enables users to define the optimal number of adjacent SNPs to be considered. In this section, we introduce a rolling-window approach that scans the IBD data. In GWAS and genome-wide IBD, SNPs in close genomic proximity often exhibit high correlation due to LD. Further, perturbations of biologically functional regions can involve multiple SNPs within a local region. By including a greater number of SNPs in the hypothesis test, it is anticipated that the valuable LD information among neighboring SNPs can be better utilized (Bao and Wang 2017). In addition, functional effects from separate variants that disrupt the same motif or gene(s) in a region can be detected. To implement this approach, we utilized Algorithm 1.

Algorithm 1. Sliding Window IBD Analysis and Permutation Test

1: **Input:**$\mathrm{IBD}\in {\left\{0,1\right\}}^{t\times m}$, No. paired subjects *t* and loci *m*

2: **Output:** IBD’s present in a rolling window: IBDW

3: **Output:** *P* values of the permutation test: P

4: *w* ← window size

5: $\mathrm{IBD}W^{t\times (m-w+1)}\leftarrow \left[\;\right]\;\&\;P^{(m-w+1)\times 1}\leftarrow \left[\;\right]$

6: **for** *j *=* *1 to m−w+1**do**

7:   **if**$\forall k\in \{0,\ldots ,w-1\},\mathrm{IB}D_{:,j+k}=1$**then**

8:     $\mathrm{IBD}W_{:,j}\leftarrow 1$

9:   **else**

10:     $\mathrm{IBD}W_{:,j}\leftarrow 0$

11:   **end if**

12:   **if** Permutation **then**

13:     $I_{\text{case},j}\leftarrow \sum_{\text{case}}\mathrm{IBD}W_{:,j}\;\;\;\&\;\;\;I_{\text{cont},j}\leftarrow \sum_{\text{cont}}\mathrm{IBD}W_{:,j}$

14:     $D_{j}\leftarrow I_{\text{case},j}-I_{\text{cont},j}$

15:     *n_p_* ← No. permutations

16:     **for** *p *=* *1 to *n_p_* **do**

17:      permute labels and compute $D_{j}^{\left(p\right)}$

18:     **end for**

19:     $P_{j}\leftarrow \frac{\sum_{p=1}^{P}I(D_{j}^{\left(p\right)}\geq D_{j})}{n_{p}}$

20:   **end if**

21: **end for**

22: **Return:**IBDW and P

The output of the analysis is a data frame that contains the *P* values corresponding to each window. To facilitate the visualization and exploration of these results, we utilize the plot() method, which serves as a wrapper for the plotly package. This integration enables the generation of interactive plots that offer an engaging and dynamic way to interact with the *P* values results similar to Fig. 1d.

### 2.4 Haplotype block extraction as a tool for association testing

The pipeline also offers the functionality to extract haplotype sequences within the sliding windows (Fig. 1e). This capability is based on the observation that segments of haplotypes tend to be inherited in blocks within specific population groups due to recent recombination events. Consequently, the boundaries of these haplotype blocks and the specific haplotypes they encompass exhibit a high degree of correlation across individuals from a population. Leveraging this haplotype framework provides substantial statistical power for association studies investigating genetic variation within each region (Zhang *et al.* 2002, Clark 2004). To implement this approach, we utilized Algorithm 2.

Algorithm 2. Haplotype Block Extraction and G-Test

1: **Input:**$\mathrm{IBD}\in {\left\{0,1\right\}}^{t\times m}$, No. paired subjects *t* and loci *m*

2: **Input:** maternal/paternal haplotypes $H^{p},\;H^{m}\in {\left\{0,1\right\}}^{n\times m}$, No. subjects *n*

3: **Output:** Extracted Haplotypes: $L_{\text{case}}$ and $L_{\text{cont}}$

4: **Output:** *P* values of the G-test: P

5: *w* ← window size

6: $\mathrm{IBD}W^{t\times (m-w+1)}\leftarrow \left[\;\right]$$\;\;\;\&\;\;\;L_{\text{case}}\leftarrow \left[\;\right]$$\;\;\;\&\;\;\;L_{\text{cont}}\leftarrow \left[\;\right]$

7: **for** *j *=* *1 to m−w+1**do**

8:   **if**$\forall k\in \{0,\ldots ,w-1\},\mathrm{IB}D_{:,j+k}=1$**then**

9:     $\mathrm{IBD}W_{:,j}\leftarrow 1$

10:   **else**

11:     $\mathrm{IBD}W_{:,j}\leftarrow 0$

12:   **end if**

13:   **if**$\mathrm{IBD}W_{:,j}=1$**then** ▹ concatenate extracted segments

14:     $L_{\text{case}}^{\left[j\right]}\leftarrow \left[\begin{array}{c} H_{\text{case},j:j+w-1}^{p}\;H_{\text{case},j:j+w-1}^{m} \end{array}\right]$

15:     $L_{\text{cont}}^{\left[j\right]}\leftarrow \left[\begin{array}{c} H_{\text{cont},j:j+w-1}^{p}\;H_{\text{cont},j:j+w-1}^{m} \end{array}\right]$

16:   **end if**

17:   **if** G-Test **then**

18:     $E_{ij}\leftarrow$ expected count of *j*th haplotype sequence in *i*th group (case/control) under the null

19:     $O_{ij}\leftarrow$ observed count of *j*th haplotype sequence in *i*th group (case/control)

20:     $G\leftarrow 2\sum_{i,j}O_{ij}\;\ln\left(\frac{O_{ij}}{E_{ij}}\right)$

21:     df← total No. haplotype segments - 1

22:     $P_{j}\leftarrow P(\chi^{2}\geq G|df)$

23:   **end if**

24: **end for**

25: **Return:**$L_{\text{case}},$$L_{\text{cont}}$ and P

The gwid package can identify subjects with a specific haplotype at a given location when an IBD region is detected using haplotype_structure() function. However, it cannot determine haplotype boundaries based on the decay of LD across blocks, as haplotype selection relies on a fixed window size. The gtest() method facilitates a log-likelihood ratio *G*-test and permutation test to analyse the frequency of these haplotypes across various groups, yielding *P* values for further assessment.

### 2.5 Shiny application

The package gwid offers a user-friendly Shiny application designed to analyse and visualize data effectively. When users input the location of their data files into the launch_app() function, the Shiny application initiates. Within this application, users can seamlessly navigate and select specific datasets or chromosomes and genomic locations, as well as the desired type of analysis. The results are displayed as plots, and there is an option for users to download these data results. The critical advantage of using Shiny in this context lies in its ability to substantially decrease the time required for analysis. This efficiency enables users to obtain results rapidly without the need for extensive programming skills.

## 3 Application

We demonstrated the key functionalities of our package using the rheumatoid arthritis (RA) GWAS dataset. This dataset consisted of DNA samples collected from 478 individuals diagnosed with RA and a control group of 1434 individuals without RA. The control group is downsampled into three groups of 478 samples each to evaluate the robustness and replicability of the findings. Genotyping was performed using the Illumina Infinium array. All samples were obtained from a genetically homogeneous population in central Wisconsin exhibiting elevated relatedness structure(McCarty *et al.* 2005). The final analysis considered a total of 336 185 autosomal single nucleotide polymorphisms (SNPs).

To evaluate the presence and extent of shared identical by descent (IBD) segments, we employed the Refined-IBD software (Browning and Browning 2013, Browning *et al.* 2018, 2021). The gwid package was utilized to calculate the IBD sharing frequencies between pairwise individuals, both within the case and control groups, at each locus. The Fisher’s exact test was then applied to assess the association and linkage between the identified causal loci and the target RA trait.

Figure 1 presents the results of the analysis conducted for the IBD mapping of RA traits. Notably, we discovered a significant and previously unknown susceptibility region for RA on chromosome 3. This finding highlights the potential relevance of this chromosomal region in the development or manifestation of RA. To analyse this dataset, the Shiny web application can be utilized at https://tinyurl.com/gwid-shiny.

For detailed instructions on how to effectively utilize the package for visualization purposes, investigators can consult the vignette at http://tinyurl.com/gwid-tutorial. This resource provides comprehensive guidance and informative guidelines to support users in utilizing the package to its full potential.

For a systematic comparison of standard SNP association testing with pairwise IBD testing, Browning and Thompson (2012) provide an in-depth simulation study. This comprehensive analysis offers valuable insights into the performance and differences between these two methodologies.

## Data Availability

The data underlying this article are available on GitHub at (https://github.com/soroushmdg/gwid).
